# Establishment and characterization of a new intrahepatic cholangiocarcinoma cell line derived from a Chinese patient

**DOI:** 10.1186/s12935-022-02840-3

**Published:** 2022-12-28

**Authors:** Xin Miao, Jinjing Hu, Changpeng Chai, Huan Tang, Zhenjie Zhao, Wei Luo, Wence Zhou, Hao Xu

**Affiliations:** 1 State Key Laboratory of Veterinary Etiological Biology, Key Laboratory of Animal Virology of the Ministry of Agriculture, Lanzhou Veterinary Research Institute, Chinese Academy of Agricultural Sciences, Lanzhou, 730000 China; 2 The Forth Department of General Surgery, The First Hospital of Lanzhou University, No. 1, Donggang West Road, Lanzhou, 730000 Gansu China; 3 Department of General Surgery, The Second Hospital of Lanzhou University, Lanzhou, 730000 China; 4 The Second Clinical Medical College, Lanzhou University, Lanzhou, 730000 China

**Keywords:** Intrahepatic cholangiocarcinoma, Cell line, Establishment, Identification, Drug resistance

## Abstract

Patients with intrahepatic cholangiocarcinoma (ICC) require chemotherapy due to late detection, rapid disease progression, and low surgical resection rate. Tumor cell lines are extremely important in cancer research for drug discovery and development. Here, we established and characterized a new intrahepatic cholangiocarcinoma cell line, ICC-X1. STR testing confirmed the absence of cross-contamination and high similarity to the original tissue. ICC-X1 exhibited typical epithelial morphology and formed tumor spheres in the suspension culture. The population doubling time was approximately 48 h. The cell line had a complex hypotriploid karyotype. The cell line exhibited a strong migration ability in vitro and cell inoculation into BALB/c nude mice led to the formation of xenografts. Additionally, ICC-X1 cells were sensitive to gemcitabine and paclitaxel but resistant to 5-fluorouracil and oxaliplatin. RNA sequencing revealed that the upregulated cancer-related genes were mainly enriched in several signaling pathways, including the TNF signaling pathway, NOD-like receptor signaling pathway, and NF-κB signaling pathway. The downregulated cancer-related genes were mainly enriched in the Rap1 signaling pathway and Hippo signaling pathway among other pathways. In conclusion, we have created a new ICC cell line derived from Chinese patients. This cell line can be used as a preclinical model to study ICC, specifically tumor metastasis and drug resistance mechanisms.

**Supplementary Information:**

The online version contains supplementary material available at 10.1186/s12935-022-02840-3.

## Introduction

Intrahepatic cholangiocarcinoma (ICC) is a malignant tumor, which originates from the intrahepatic bile duct epithelial cells. This cancer ranks second in occurrence to hepatocellular carcinoma and accounts for 10–15% of all cases of primary liver cancer [1]. The incidence of ICC has globally increased in the past 30 years [2]. The onset of ICC is insidious; it easily invades the organs, tissues, and nerves around the liver and lymph nodes, leading to extrahepatic distant metastases. For some patients with early ICC, liver resection has been widely performed in patients with early ICC [3]. However, even after radical resection, patients are still prone to recurrence and metastasis. The 5-year overall survival rate of patients after surgery ranges from 25 to 40%, and the prognosis is far worse than that of hepatocellular carcinoma [3–7].

The majority of patients are hard to resect or develop distant metastases because of local tumor infiltration; therefore, treatment options other than surgery are required. Currently, gemcitabine-based chemotherapy combination regimens are used as first-line treatments for patients with unresectable or recurrent and metastatic cholangiocarcinoma. However, these regimens have limited effect on improving the long-term survival of patients [8].

The choice of an appropriate course of treatment for patients and improving their survival requires a thorough understanding of the pathogenesis of ICC, the complex interactions between the pathogenic genes, and the tumor microenvironment.

Tumor-derived cell lines provide important cell experimental models for basic and clinical research on human tumors. However, with an increase in the number of passages in vitro, some unique biological characteristics of the cell line gradually change or disappear and even characteristics that the original cell does not have may be produced. Cell cross-contamination has also been detected in many cell lines. HeLa, T-24, and M14 are the most common cross-contaminating cell lines; therefore, studies using these cell lines may produce erroneous results [9–12].

In the United States, the lack of reproducibility of preclinical studies costs about $56 billion annually. The cost of biological reagents and reference materials accounts for about one-third of the total cost. Several studies are not reproducible because of the misidentification, contamination, genetic drift, and clonal evolution of cell lines, thereby hindering cancer research [13].

ICC is a highly heterogeneous disease with multimolecular profiles and diverse clinical outcomes. Tumor heterogeneity hinders patient prognosis and treatment. Notably, the lack of availability of ethnically diverse cell lines has impeded the development of drugs beneficial to patients of different ethnicities. Therefore, ethnic differences should also be factored into tumor research [14–18].

At present, there are many ICC cell lines available for research, but the number of ICC cell lines of Chinese origin is very small. This is incompatible with China’s huge population. To further study the characteristics of ICC in the Chinese population, more Chinese-derived ICC cell lines need to be established. The new ICC cell lines can enrich the existing cell line bank and can provide a preclinical model for the development of new drugs to treat patients with ICC [19].

In this study, we established a new ICC cell line of Chinese origin and named it ICC-X1. Further, we determined its biological and molecular characteristics. Our model can be used as an effective tool to study the mechanisms underlying the occurrence, development, metastasis, and drug resistance of ICC.

## Materials and methods

### Acquisition of specimens

ICC tissues were obtained from surgical specimens resected in the Fourth Department of General Surgery, the First Hospital of Lanzhou University, China. The patients did not receive any preoperative treatment. Fresh ICC tissue specimens were cut under sterile conditions and transported to the laboratory for primary cell isolation and culture in the shortest possible time. The postoperative pathology report revealed a poorly differentiated ICC in the specimens. This study was approved by the Ethics Committee of the First Hospital of Lanzhou University (Approval number: LDYYLL2022-345). Informed consent was obtained from the patient.

### BALB/C nude mice

Six-week-old sterile BALB/C female nude mice were purchased from Changzhou Cavens Laboratory Animal Co., Ltd (Changzhou, China) and raised in the Animal Experiment Center of Lanzhou University, China. Mice were provided ad libitum access to sterile water and food. Animal experiments were carried out per the requirements of the Laboratory Animal Protection Committee of Lanzhou University, China. When an animal became seriously ill during the experiment, it was euthanized by carbon dioxide exposure followed by cervical dislocation.

### Establishment of cell lines

The freshly obtained tissue samples were placed on an ultraclean bench and rinsed three times with phosphate-buffered saline (PBS) at 4 °C; the blood vessels, necrotic tissues, and interstitial tissues on the surface were removed using sterile forceps. The tissue sample was finely chopped and incubated with collagenase type II and neutral protease solution at 37 °C for approximately 2 h in a humidified atmosphere containing 5% CO_2_. The digested solution was filtered using a 200-mesh sieve and the filtrate was centrifuged at 300×*g* for 3 min. The supernatant was discarded, the pellet was resuspended in 5 mL PBS, and centrifuged again at 300×*g* for 3 min. The final pellet was resuspended in the RPMI-1640 medium containing 10% fetal bovine serum (Biological Industries Ltd., Haemek, Israel), 1% penicillin–streptomycin. The suspension was inoculated into culture plates and incubated at 37 °C in a 95% air and 5% CO_2_ atmosphere. The medium was changed twice a week. When the cells reached 70–80% confluence, cell passage and cryopreservation were performed under optimal conditions.

### Control cell line

The human cholangiocarcinoma RBE cell line was obtained from the Cell Bank of Type Culture Collection of the Chinese Academy of Sciences, Shanghai, China in 2016. The cell line was cultured in RPMI-1640 medium supplemented with 10% fetal bovine serum (Biological Industries Ltd.), 100 U/mL penicillin, and 100 µg/mL streptavidin-mycin (Biological Industries Ltd.).

### Morphology of the ICC-X1 cells

ICC-X1 cells were seeded in the 6-well plates and incubated at 37 °C in a humidified atmosphere containing 5% CO_2_ for 2 weeks. The cells were harvested every 24 h and analyzed using an inverted microscope to observe their general morphology.

### Short tandem repeat (STR) detection

Fifteen passages of logarithmic growth phase ICC-X1 cells were used for these experiments. STR analysis was performed according to the protocols published by the American National Standard Institute (ANSI/ATCC ASN-0002-2011 Authentication of Human Cell Lines: Standardization of STR Profiling).

### Karyotypic analysis

Chromosomal analysis was performed on the cells at passage. The cells were treated with 0.1 µg/mL colchicine for 1 h and were digested with trypsin/EDTA; slides were prepared according to standard methods. The hypotonic treatment was performed using 0.075 M potassium chloride solution for 20 min at room temperature. The slides with fixed cells were stained using trypsin-Giemsa to identify individual metaphase chromosomes. Fifty chromosomes in the split phase were randomly counted under an oil-immersion objective lens (1000× magnification) of a light microscope and 10 well-dispersed G passage specimens were selected for karyotypic analysis. Abnormal chromosomes were identified according to the International Human Cytogenetic Nomenclature System, 2016 [20].

### Quantification of population doubling time

Forty passages of logarithmic growth phase cells were used for these experiments. Tumor cells in the logarithmic growth phase were digested with trypsin and a single-cell suspension containing 1000 cells/100 μL was prepared. The cells were seeded into 96-well plates at 1000 cells/well and incubated at 37 °C with 5% CO_2_ and saturated humidity. The cell doubling time was calculated after 24 h. The cells were incubated with a Cell Counting Kit-8 (CCK-8; Dojindo Laboratories, Kumamoto, Japan) for 2.5 h, and absorbance was measured at a wavelength of 450 nm. The cell doubling time was calculated using the formula: Td = t × lg2/lg(N1/N0), where Td is doubling time, T is the time interval, N1 is the endpoint cell number, and N0 is the initial cell number. The growth curve was drawn with the time on the X-axis and the absorbance value on the Y-axis.

### Ultrastructure of the ICC-X1 cells

Thirty-five passages of logarithmic growth phase ICC-X1 cells were used for these experiments. After washing the cell slides with physiological saline, they were quickly put into 4% glutaraldehyde (SPI-CHEM, USA) in a fixative solution and rinsed three times with phosphate buffer. The cells were dehydrated with 50%, 70%, 80%, 90%, and 100% *tert*-butanol in a step-by-step gradient (5 min at each concentration). Next, the cells were put into the JEOL JFD-320 cold dryer, and the dried sample was taken out when the temperature equilibrated to room temperature. A conductive paste was used to coat the sample holder with a JEOL JFCC-160 ion sputterer. The specimens were observed and photographed using a scanning electron microscope (HITACHI Regulus 8100).

### Transwell chamber migration assay

Thirty passages of logarithmic growth phase cells were used for experiments. The lower chamber was filled with 600 μL of culture medium containing 5% fetal bovine serum and the upper chamber was filled with 1 × 10^5^ cells/well of RBE and ICC-X1; the chambers were then placed in the corresponding lower chambers. After culturing for 8 h, the chamber was taken out, and the cells in the chamber were wiped off and placed in 0.1% crystal violet staining solution for 30 min. The cells passing through the membrane were observed under a light microscope; five fields of view were randomly selected and the cells passing through the membrane were counted.

### Plate colony formation assay

ICC-X1 cells of passage 45 and RBE cells in the logarithmic growth phase were digested with 0.25% trypsin. Next, single cells were pipetted into the RPMI-1640 medium supplemented with 10% fetal bovine serum for further use. The cell suspension was diluted in gradient times, and 500 cells/well were inoculated into a 6-well plate. The evenly dispersed cells were then incubated at 37 °C with 5% CO_2_ and saturated humidity for 2 weeks. The cultures were observed periodically and terminated when macroscopic clones appear in the petri dish. The number of clones with more than 10 cells under the microscope was counted. The colony formation rate (%) was calculated using the formula: (number of clones/number of inoculated cells) × 100.

### Spheroid formation assay

Logarithmically growing cells of passage 50 were collected, trypsinized, and cultured in serum-free conditioned stem cell medium (RPMI-1640 medium supplemented with 1 × B27, 20 ng/mL human EGF, 10 ng/mL human FGF, 0.4% bovine serum albumin, and 4 µg/mL insulin). The cultured cells were inoculated (1.5 × 10^5^ cells/well) into a 6-well plate (Ultra-low adherent plate). Spheroid formation was monitored on days 7, 10, and 14 after seeding.

### Organoid culture

The logarithmically growing cells of passage 40 were collected, trypsinized, and prepared as a single-cell suspension. The tumor cell concentration was adjusted to 1000 cells/100 μL. A volume of 100 μL of the cell suspension was taken, mixed with matrigel 1:1, inoculated into a 12-well plate, and incubated at 37 °C for 30 min to form a gel. To each well, 1 mL of organoid culture medium [RPMI-1640 supplemented with 1% Glutamax (Invitrogen), 1% penicillin/streptomycin, 1× B27 supplement (Gibco), 50 ng/mL EGF (Peprotech), 1.25 mM *N*-acetylcysteine (Sigma), 10 nM gastrin (Sigma), 10 mM nicotinamide (Sigma), Rspo-1 (Peprotech), 5 mM A83-01 (MCE) and 10 mM Y-27632 (MCE)] was added, and the cell culture plate was placed at 37 °C and humidity in an incubator with 5% CO_2_. The culture medium was changed every 3–4 days and passaged every 1–2 weeks.

### Drugs

Gemcitabine was purchased from Jiangsu Hansoh Pharmaceutical Group Co., Ltd., China; oxaliplatin was purchased from Jiangsu Hengrui Pharmaceutical Co., Ltd., China; 5-fluorouracil (5-FU) was purchased from Tianjin Jinyao Pharmaceutical Co., Ltd., China; and paclitaxel purchased was from Jiangsu Osaikang Pharmaceutical Co., Ltd. Gemcitabine, 5-FU, and paclitaxel were dissolved in normal saline and oxaliplatin was dissolved in 5% glucose solution. The stock solutions were aliquoted as different working solutions to avoid degradation.

### Drug sensitivity test

Logarithmically growing RBE and ICC-X1 cells of passage 50 were collected separately and prepared as single-cell suspensions after trypsinization. The cells were inoculated into 96-well plates at 10,000 cells/well, and each group was inoculated into 6 wells. After cell adherence, different concentrations of antitumor drugs were added to the experimental group and equal volumes of solvent of each drug group were added to the vehicle control group. After 72 h of incubation, the complete medium was replaced with 100 μL of serum-free medium containing 10% CCK-8 reagent. The optical density was measured at 450 nm after 2 h.

### Tumorigenicity in BALB/C nude mice

The cells were prepared at passage 25 to determine their tumorigenicity in BALB/C nude mice. The cultured cells (1 × 10^7^ cells/mL) were collected, washed, resuspended in 0.1 mL of complete RPMI-1640 medium, and injected subcutaneously into the middle and posterior left axilla of three 6-week-old female BALB/C nude mice. Tumor diameters and body weight were measured every week after cell injection and tumor volume was calculated as ½ × length × (width^2^) [21]. Tumor-bearing mice were sacrificed after 4 weeks. Tumor tissue was excised and fixed with 10% formalin, and routine histopathological and immunohistochemical examinations were performed.

### Immunohistochemistry

Cells at passage 36 were digested and grown on sterile glass slides. At 48 h, slides were washed with PBS, fixed with 4% paraformaldehyde for 15 min, air-dried, and treated with 0.5% Triton X-100 for 20 min. For primary tumor tissues, transplanted tumors, and organoids, the specimens were immersed in a formaldehyde solution for fixation, dehydration, embedding, and sectioning. The slides were then covered with the following antibodies: mouse mAb against human cytokeratin (CK)7 (1:300; Servicebio), mouse mAb against human CK19 (1:1000; Servicebio), rabbit mAb against Ki-67 (1:200; Servicebio), mouse mAb against human p53 clonal antibodies (1:200; Servicebio), mouse mAb against human E-cadherin (1:600; Servicebio), and mouse mAb against human vimentin (1:1000; Servicebio). The slides were incubated with the antibodies for 60 min and washed thoroughly with PBS. Biotinylated rabbit anti-mouse IgG (1:200; Servicebio) was then added for 15–20 min and the slides were washed. Then, a 3,3′-diaminobenzidine solution was added to the slides for 1–5 min at room temperature. Finally, slides were rinsed with distilled water, stained with hematoxylin and eosin (H&E), and examined under a light microscope.

### Transcriptomic analysis

The mRNA profiles of ICC-X1 cells were compared to that of RBE cells to better understand their molecular features and altered pathways. First, the purity, concentration, and integrity of the extracted RNA samples were determined to ensure that qualified samples are used for transcriptome sequencing. The library was constructed using qualified samples by (1) enriching eukaryotic mRNA with magnetic beads with oligo(dT); (2) adding fragmentation buffer to randomly interrupt mRNA; (3) using mRNA as a template and synthesizing, the first cDNA chain with random hexamers and the second cDNA chain by adding buffer, dNTPs, RNase H, and DNA polymerase I, and purifying the cDNA using AMPure XP beads; (4) subjecting the purified double-stranded cDNA to end repair, adding a tail, and connecting a sequencing adapter, and then using AMPure XP beads to select fragment sizes; and (5) obtaining a cDNA library by PCR enrichment. After the library was constructed, quantitative PCR was used to accurately quantify the effective concentration of the library (library effective concentration > 2 nM) to ensure the quality of the library. After passing the library check, sequencing was performed on the Illumina platform (Illumina, Inc., USA). This project used the reference genome: Homo_sapiens.GRCh38_release95.genome.fa as a reference for sequence alignment and subsequent analysis. The reads on the StringTie alignment were used for assembly and quantification after the alignment analysis was completed.

### Statistical analysis

The statistical significance between the experimental and control group was determined using the Student’s t-test. The statistical significance was set at p ≤ 0.05.

## Results

### Establishment of ICC-X1 cell line

**Fig. 1:**
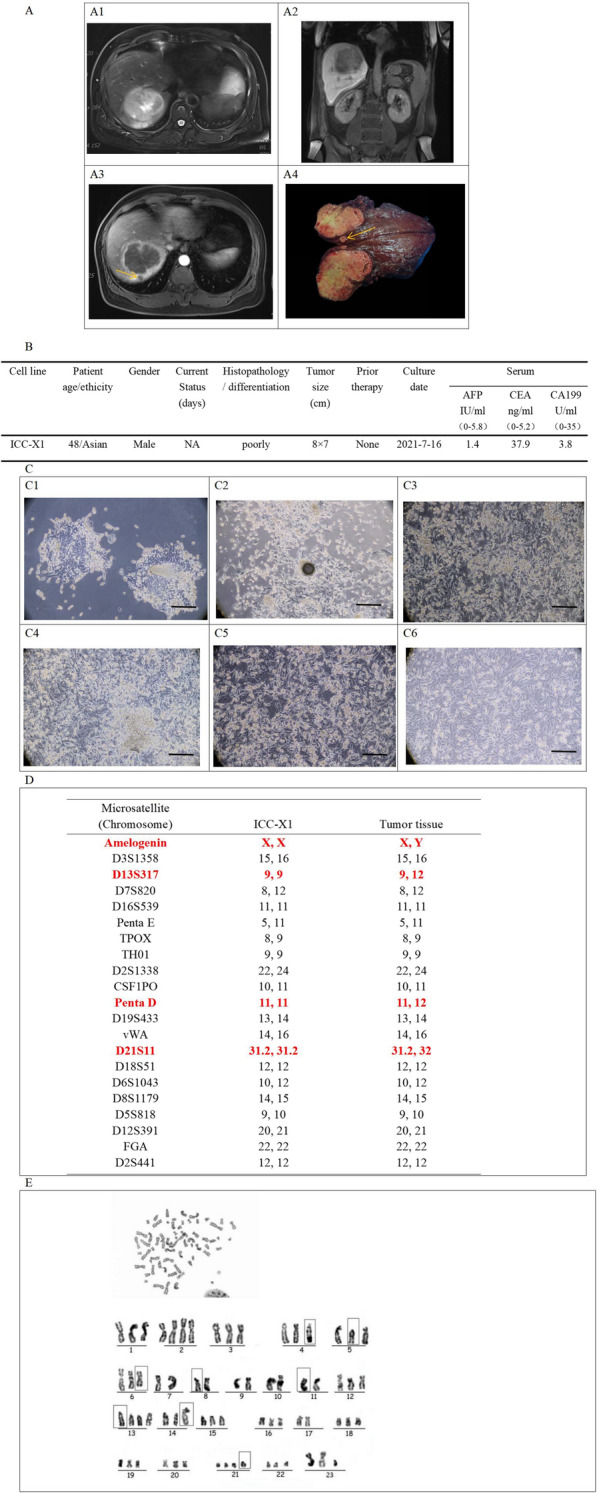
Clinical data, Morphology, Short tandem repeat (STR) detection and Karyotypic analysis of the ICC-X1 cell line. **A1**–**A3** Show an 8 × 7 cm tumor in the S7 segment of the liver, with limited diffusion, obvious enhancement in the arterial phase, and decreased enhancement in the portal venous phase and delayed phase; **A3** A satellite lesion (short arrow) can be seen next to the tumor. **A4** There was an 8 × 7 cm gray-white, ductile mass in the section of the surgically resected specimen, and a satellite lesion (short arrow) can be seen next to it. **B** Clinical and pathological profile of the patient with ICC. **C1** Shows an image of day 3 of the primary culture. **C2** Shows an image of day 7 of the primary culture. **C3** Shows an image of passage 1 of the ICC-X1 cells. **C4** Shows an image of passage 5 of the ICC-X1 cells. **C5** Shows an image of passage 15 of the ICC-X1 cells. **C6** Shows an image of thawed passage 50 of the ICC-X1 cells. Scale bars, 50 μm. **D** Comparison of genotyping results in tumor specimens obtained from patients, and ICC-X1cells at passage 10. The patient’s tumor specimen and cultured ICC-X1 cells demonstrated differentially typed loci in D21S11, AMEL, D13S317, and PentaD. **E** The karyotype of the ICC-X1 cell line shows abnormalities in both the number and structure of chromosomes

We successfully established a tumor cell line from a 48-year-old Chinese male patient with ICC in situ and named it ICC-X1. The cell line has now been passed down to passage 104. The clinical and pathological features of the tumor tissues are shown in Fig. 1A, B. The cells appeared typical epithelioid with short spindle-shaped and polygonal shapes under the inverted microscope (Fig. 1C2). With sufficient nutrition, the cells grew in layers and lost the characteristics of cell-to-cell contact inhibition (Fig. 1C4). The ICC-X1 cell line had high viability under cryopreservation with no change in the characteristics of the cells at different passages (Fig. 1C1–C6).

### STR DNA profiling and karyotypic analysis

The genetic characteristics of the ICC-X1 cells were analyzed using DNA fingerprinting. This analysis included 21 STR loci validated at different chromosomal locations. The results showed that the cultured cell line was 91.4% similar to the original tumor tissue at the 21 loci tested and did not correspond to any cells in the public cell bank (Fig. 1D and Additional file 1). Karyotypic analysis of ICC-X1 using the G-banding technique showed that ICC-X1 was mainly hypotriploid, and structural chromosomal aberrations were detected. The representative karyotype was 66, XXY del(4)(p14)del(5)(p12)del(6)(q24)del(8)(p23)der(11)der(13)rob(14:21)der(21) (Fig. 1E).

### Doubling time, ultrastructure, transwell chamber migration assay, and plate colony formation assay

**Fig. 2:**
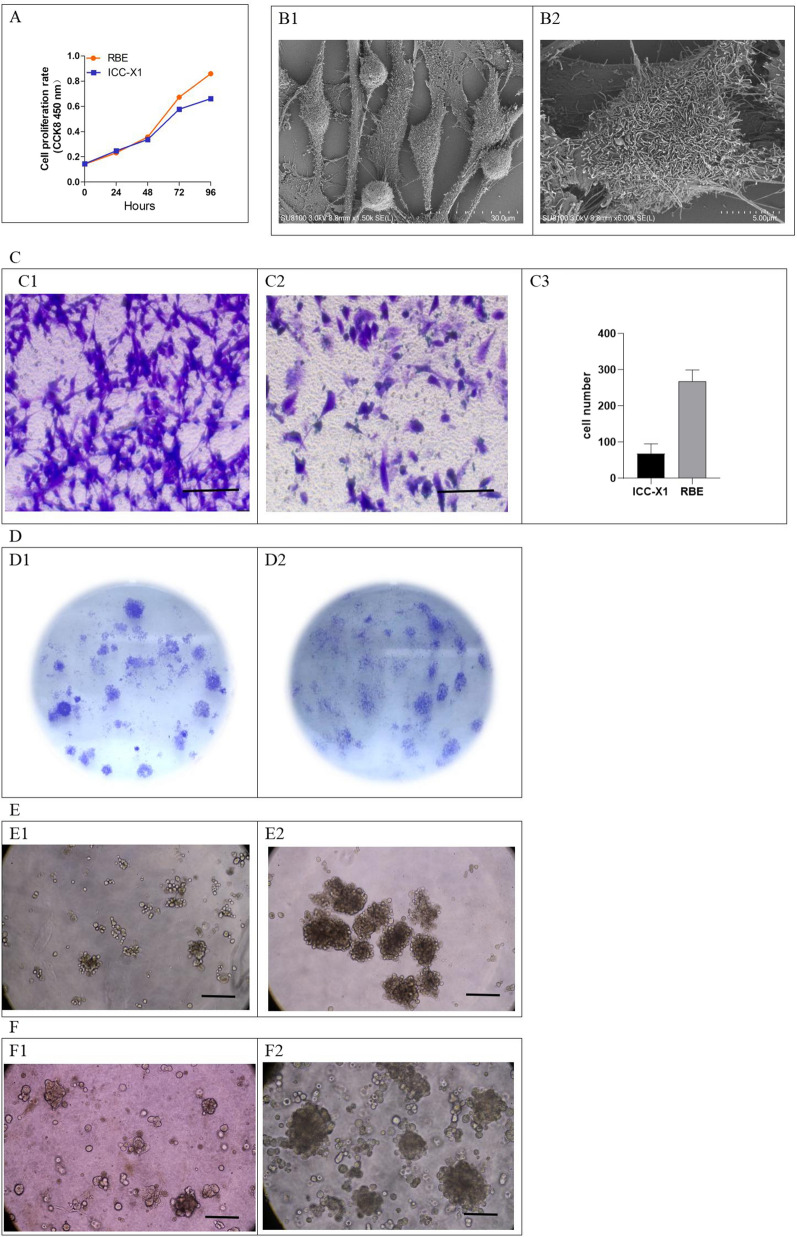
Population doubling time, Ultrastructure, cell migration ability, plate colony formation ability, Spheroid and organoid culture of the ICC-X1 cells. **A** The growth curve of the RBE and ICC-X1 cells. **B** Ultrastructure of ICC-X1 cells. **C** Difference in migratory ability between the RBE (**C1**) and ICC-X1 cells (**C2**). Scale bars, 50 μm. **D** The plate clone forming ability of the RBE cells (**D1**) and ICC-X1 cells (**D2**). **E** The spheroid culture process of ICC-X1: **E1** cultured for 1 week; **E2** cultured for 2 weeks. Scale bars, 100 μm. **F** ICC-X1 organoid culture process: **F1** cultured for 1 week; **F2** cultured for 2 weeks. Scale bars, 100 μm

The doubling times of the ICC-X1 and RBE cells were approximately 48 h (Fig. 2A).

Under the scanning electron microscope, spindle-shaped ICC-X1 cells grew well. Dense junctions were observed between cells. Some of the cells had extended filopodia and lamellipodia, showing strong invasive ability. A large number of microvilli were observed on the cell surface and their shape was relatively regular (Fig. 2B).

The cell migration ability of ICC-X1 cells was weaker than that of RBE cells (Fig. 2C). ICC-X1 and RBE cells were able to form cell clones of different sizes on the plate. The clone formation rate of ICC-X1 and RBE cells was 7.6% and 9.4% within 2 weeks, respectively (Fig. 2D).

### Spheroid formation assay and organoid culture

ICC-X1 cells were able to grow in an anchorage-free manner and produced large numbers of well-structured tumor spheres in low attachment conditions and stem cell-free serum medium, indicating a strong stemness characteristic (Fig. 2E). When ICC-X1 cells were inoculated into matrigel, the cells rapidly formed ICC organoids of various sizes approximately in a week (Fig. 2F).

### Sensitivity to chemotherapeutic drugs

**Fig. 3:**
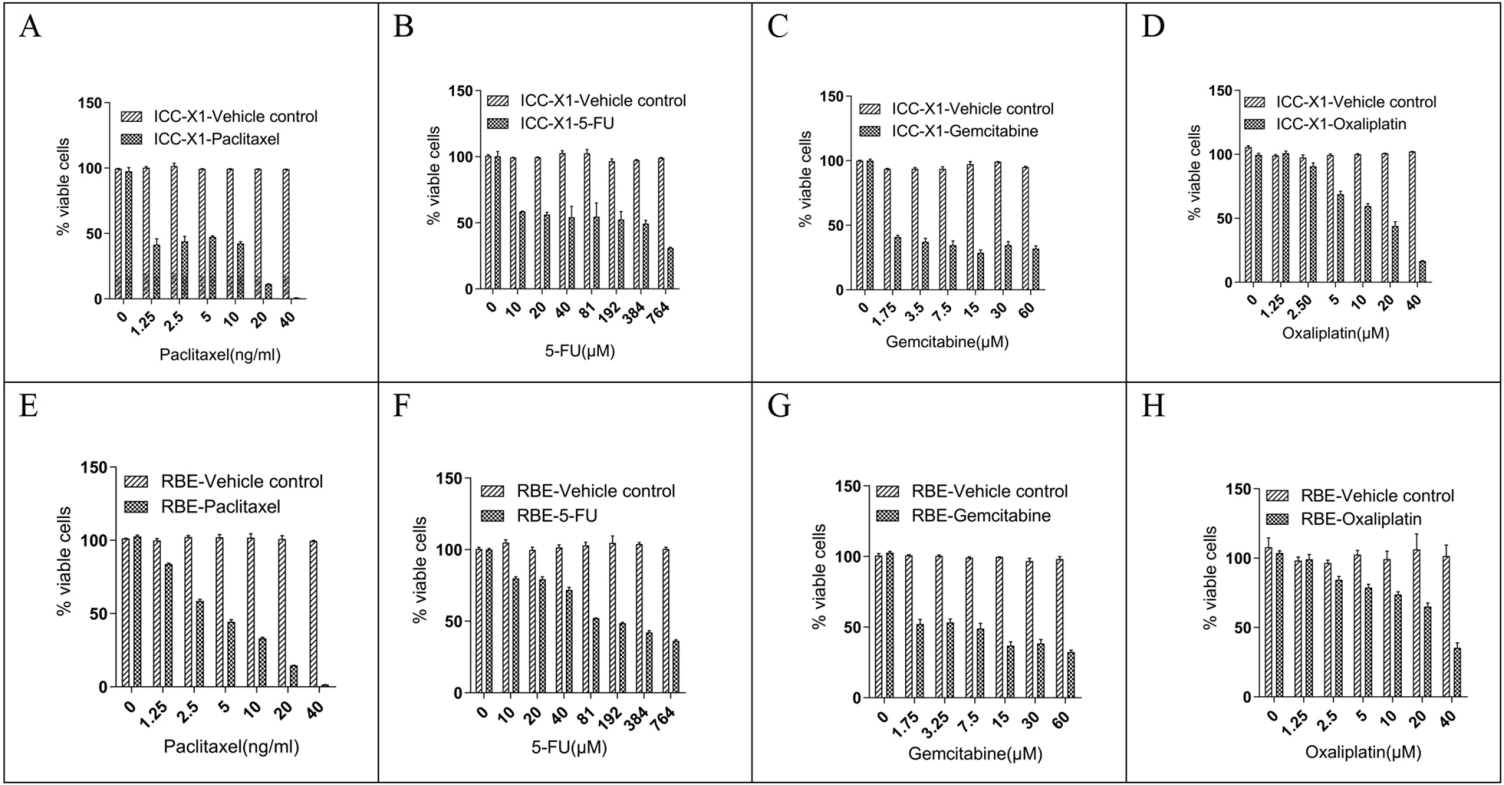
Drug sensitivity of the RBE and ICC-X1 cells. Dose–effect graphs of paclitaxel (**A**, **E**), gemcitabine (**B**, **F**), 5-FU (**C**, **G**), and oxaliplatin (**D**, **H**) in the ICC-X1 and RBE cells

We evaluated the sensitivity of RBE and ICC-X1 cells to paclitaxel, 5-FU, oxaliplatin, and gemcitabine. ICC-X1 cells were sensitive to gemcitabine and paclitaxel but resistant to 5-FU and oxaliplatin (Additional file 2). The RBE cells showed resistance to paclitaxel, 5-FU, oxaliplatin, and gemcitabine (Fig. 3).

### Tumorigenicity in BALB/c nude mice

**Fig. 4:**
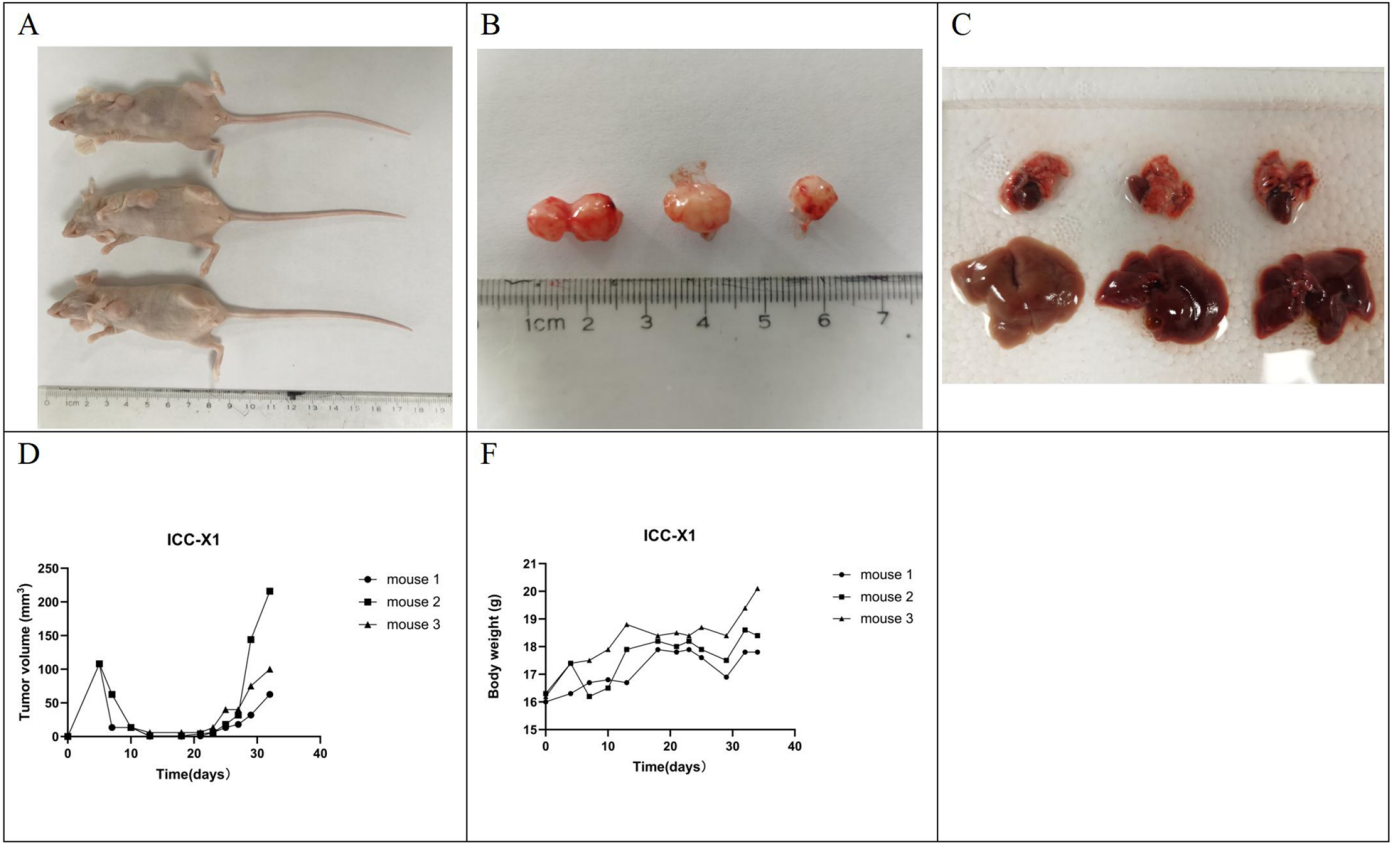
Tumorigenicity in the BALB/C nude mice. **A**, **B** ICC-X1 rapidly formed xenografts after inoculation into the BALB/C nude mice. **C** No metastatic lesions were found in the lung and liver tissues of the BALB/C nude mice. **D**, **E** Growth curve of the transplanted tumor

We performed xenograft tumor formation experiments in three nude mice and monitored tumor growth every week to verify the tumorigenicity of ICC-X1 cells in nude mice. Transplantation tumors were observed in all three mice within 4 weeks, and no metastases were found in the lungs and livers of the nude mice after dissection (Fig. 4).

### Histopathological analysis and immunophenotyping

**Fig. 5:**
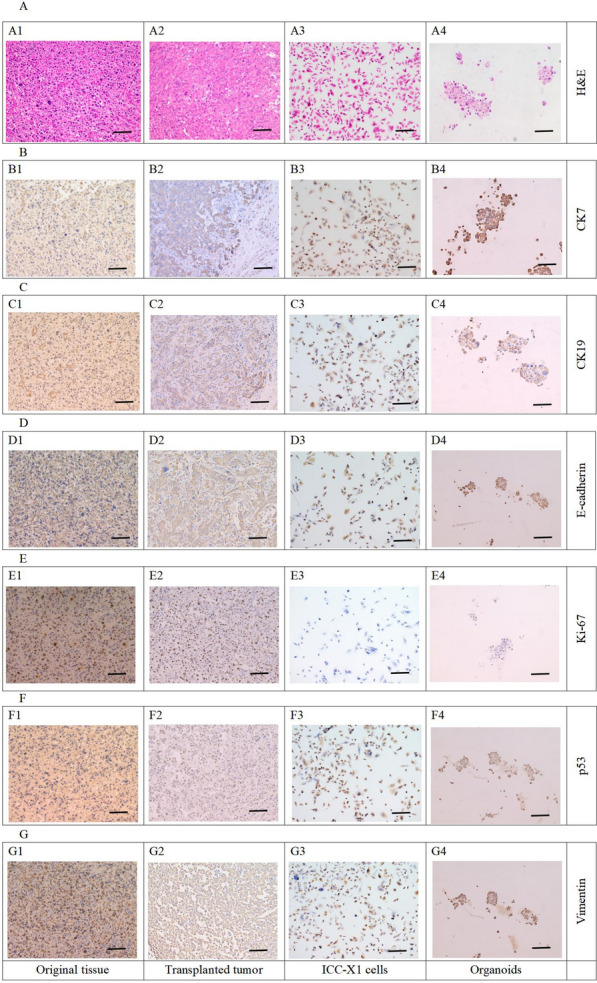
HE staining and Immunohistochemical staining of the primary tissue, transplanted tumor, ICC-X1 cells, and organoids. **A** HE staining of the original tissue, transplanted tumor, ICC-X1 cells and organoids. **B** CK7 staining of the original tissue, transplanted tumor, ICC-X1 cells and organoids. **C** CK19 staining of the original tissue, transplanted tumor, ICC-X1 cells and organoids. **D** E-cadherin staining of the original tissue, transplanted tumor, ICC-X1 cells and organoids. **E** Ki-67 stainingof the original tissue, transplanted tumor, ICC-X1 cells and organoids. **F** p53 staining of the original tissue, transplanted tumor, ICC-X1 cells and organoids. **G** Vimentin staining of the original tissue, transplanted tumor, ICC-X1 cells and organoids. Scale bars, 50 μm

We performed the histopathological analysis of primary tumor tissues, xenografts, organoids, and ICC-X1 cells. H&E staining showed that the primary tumor xenografts and organoids were poorly differentiated (Fig. 5A).

Immunohistochemical staining revealed that CK7, CK19, E-cadherin, p53, Ki-67, and vimentin were positive in ICC-X1 cells, and a high degree of similarity was observed in the expression CK7, CK19, E-cadherin, p53, Ki-67, and vimentin in primary tumor tissues, transplanted tumors, ICC-X1 cells, and organoids (Fig. 5B–G).

### Transcriptomic analysis

**Fig. 6:**
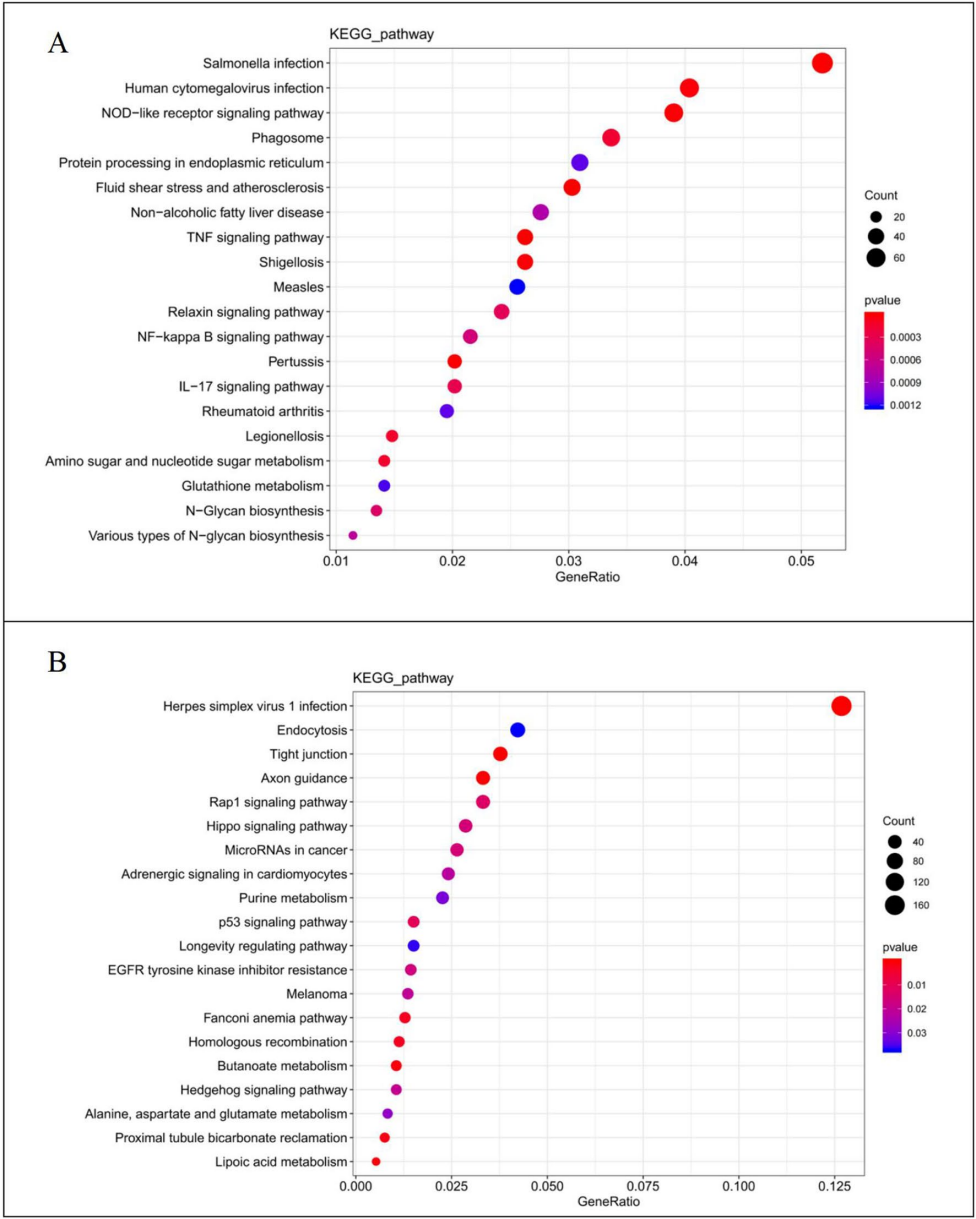
Transcriptomic profile of the ICC-X1 cells. **A** The first 20 GO biological processes were overrepresented among the upregulated transcripts (p < 0.01). **B** The first 20 GO biological processes were overrepresented among the downregulated transcripts (p < 0.01)

Transcriptomic analysis revealed 2824 upregulated genes in the ICC-X1 cells compared with RBE cells. We used the DAVID tool v6.8 (https://david.ncifcrf.gov/) for gene enrichment analysis because of the large number of differentially expressed genes. We found that the upregulated cancer-related genes were mainly enriched in several signaling pathways including the TNF signaling pathway, NOD-like receptor signaling pathway, and NF-κB signaling pathway. Among these, the TNF signaling pathway and NF-κB signaling pathway exist in many important cellular processes, such as inflammatory response, proliferation and metastasis of tumor cells, and occurrence and development of various tumors. Figure 6A presents the top 20 overrepresented biological processes with p < 0.01.

Similarly, the transcriptomic analysis revealed 2770 downregulated genes in the ICC-X1 cells. We found that the downregulated cancer-related genes were mainly enriched in the Rap1 signaling pathway and Hippo signaling pathway among others. The primary function of the Rap1 and Hippo pathways is to restrict tissue growth and regulate cell proliferation, differentiation, and migration. When these pathways are dysregulated, abnormal cell growth and tumor formation occur. Figure 6B presents the top 20 overrepresented biological processes with p < 0.01.

## Discussion

The incidence of ICC in China has been increasing in recent years [22]. The majority of patients are at an advanced or late stage of the disease when they receive treatment, and the prognosis is very poor due to the insidious onset of this disease and atypical early clinical symptoms. Radical surgical resection is currently the only possible cure for ICC; however, the postoperative recurrence rate is as high as 60 to 70% [23]. There is currently no effective medication for treating ICC because its pathogenesis is still not fully understood. New and ethnically diverse ICC cell lines can be a good experimental model to study the occurrence and development mechanism of a tumor. Such cell lines will also be instrumental in drug discovery and development.

In this study, a new ICC cell line, ICC-X1, was successfully established from a primary culture and subculture of the primary tumor tissue derived from a Chinese patient. The cell line was genetically verified using STR analysis and was found to be similar to the primary tumor tissue. It was not cross-contaminated with other tumor cell lines in public cell banks. The cell line was further characterized through doubling time determination, chromosome analysis, drug sensitivity testing, nude mouse transplantation tumor formation experiments, and cell stemness detection.

We found that the primary culture of tumor tissues having > 40% nuclear protein Ki-67 has a higher success rate. This aspect is related to the high malignancy of the tumor itself, and the rapid proliferation of tumor cells so that tumor cells are not easily competed by fibroblasts. This result was consistent with previous findings [24, 25].

Organoids are a promising disease model that can be used to better understand biology and to test the efficacy of drugs in vitro. Few studies on ICC organoids have been reported and the culture success rate of these organoids is lower than that of gastrointestinal tumor organoids [26]. In this study, we successfully established ICC organoids from the ICC-X1 cells. The organoid activity was not affected after cryopreservation and resuscitation. The ICC-X1, original tumor tissue, and organoids were verified using immunohistochemistry. The cell lines and organoids retained most of the genomic and transcriptomic functional characteristics of the primary tumor [27]. These results provide a rationale for using the ICC-X1 cell line or organoid as a preclinical model for precision medicine.

Nude mice are the experimental animals of choice to study tumor molecular mechanisms, the development of antitumor drugs, and drug sensitivity. The tumor formation rates vary greatly after the cell lines are inoculated into nude mice. The tumor formation rates of breast cancer and colon cancer cell lines are higher, whereas the transplantation rates of hepatobiliary tumor cell lines are lower. The commonly used ICC cell lines, TKKK, RBE, and HCCC-9810, are unable to form xenograft tumors after inoculation in nude mice, greatly limiting in vivo tumor research [28]. The ICC-X1 cell line had a good ability to form subcutaneously transplanted tumors in nude mice with a tumor formation rate of 100%. The incubation period for the formation of subcutaneously transplanted tumors was short; the tumor formation cycle was short. This tumor model is easy to establish with the simple experimental operation. The operation can be completed by subcutaneously injecting the cultured cells, too much pretreatment on nude mice or cell lines is not required. Moreover, the transplanted tumors grew subcutaneously for easy observation and measurement.

Intrahepatic cholangiocarcinoma can be divided into proliferative and inflammatory types based on differences in gene expression profiles and signaling pathways [29]. The proliferative type is mainly characterized by excessive activation of cell proliferation-related signaling pathways such as RAS/MAPK, MET, EGFR, ERBB2, and NOTCH; therefore, the tumor is more aggressive. The inflammatory type is characterized by the enrichment of signaling molecules such as interleukins (IL), chemokines, and continuous expression of STAT3. The prognosis of patients with inflammatory type ICC is better than that of the patients with proliferative type [29, 30]. Transcriptomic data analysis revealed that the ICC-X1 cells had upregulated expression of TNF, NOD-like receptors, NF-κB, and other inflammation-related genes and had downregulated expression of proliferation-related genes such as RAP1 and Hippo. Therefore, it is suggested that ICC-X1 cells are of inflammatory type.

TNF-α is a key cytokine involved in inflammation, immunity, cell homeostasis, and tumor progression. The role of TNF-α in cancer development is a double-edged sword. TNF-n is a cytokine that can kill tumor cells. In contrast, TNF-T can also mediate the proliferation, invasion, and metastasis of many cancer cells to promote and develop tumors [31]. In addition, TNF-3 enhances tumor angiogenesis through various angiogenic factors, including IL-6, IL-8, and VEGF [32]. TNF-3 induces Epithelial-mesenchymal transition in different cancer models and is involved in chemoresistance [33]. Simultaneously, transmembrane TNF-N can also participate in drug resistance through NTF-NF-aB mediation and other pathways [34, 35]. Furthermore, soluble TNF-N induces resistance to BRAF inhibitors in melanoma cells and cisplatin chemotherapy in malignant pleural mesothelioma [36, 37]. The high expression of TNF, NOD-like receptors, and NF-hB in ICC-X1 cells may be the reason for the resistance of ICC-XI cells to 5-FU and oxaliplatin.

Bcl-2 proteins inhibit the apoptotic process by inhibiting the release of cytochrome C from mitochondria and preventing the oxidative destruction of cells. Their overexpression and phosphorylation are related to the regulation of cell proliferation, tumor formation, and multidrug resistance. It plays an extremely important role and is an effective tumor therapy target [38–41]. Transcriptome sequencing of ICC-X1 showed downregulation of Bcl-2 expression, which may be directly related to the sensitivity of ICC-X1 to gemcitabine and paclitaxel.

RNA seq data revealed that many genes highly expressed by RBE relative to ICC-X1 are related to cell proliferation and cell cycle, whereas the ICC-X1 cell line does not express these genes that promote cancer cell proliferation. For example, overexpression of TM4SF4 significantly promotes the growth and colony formation of HCC cells [42]. ICC-X1 minimally expresses GPR87, which can promote the growth and metastasis of CD133+ cancer stem-like cells [43]. ETS homologous factor (EHF) is a member of the E26 transformation specific (ETS) transcription factor family. The knockout of EHF in ovarian cancer cells significantly inhibits cell proliferation and increases the number of G1 phase cells. After EHF knockout, proteins that promote the cell cycle (Cyclin B1, Cyclin D1, and PCNA) are downregulated, and proteins that negatively regulate cell cycle progression (P21) are upregulated [44]. LMP2A in EBV-positive gastric cancer activates EHF through STAT3 phosphorylation [45]. EHF, by activating TGF-b1 transcription and typical TGF-a signal transduction, promotes colorectal cancer progression [46]. EHF is hardly expressed in ICC-X1 cells. The proliferation and migration ability of ICC-X1 is far lower than that of RBE cells, which may be caused by the nonexpression or extremely low expression of these genes in ICC-X1.

## Conclusions

We have established and characterized ICC-X1, a new ICC cell line derived from the primary tumor of a Chinese patient. This cell line can be used as a resource to study the pathogenesis, invasion, and metastasis mechanisms of ICC. Further, this cell line could be a potential preclinical model for drug discovery and development to diagnose and treat patients with ICC.

## Supplementary Information

**Additional file 1**. DNA fingerprinting of ICC-X1 cells.

**Additional file 2**. The dose-response curve of ICC-X1.

## Data Availability

The datasets used and/or analysed during the current study are available from the corresponding author upon reasonable request.
