# Supplementary figures and images for: Establishment and characterization of a new intrahepatic cholangiocarcinoma cell line derived from a Chinese patient
Source: Cancer Cell Int. 2022 Dec 28;22:418. doi: 10.1186/s12935-022-02840-3 (PMC9795767; doi:10.1186/s12935-022-02840-3)

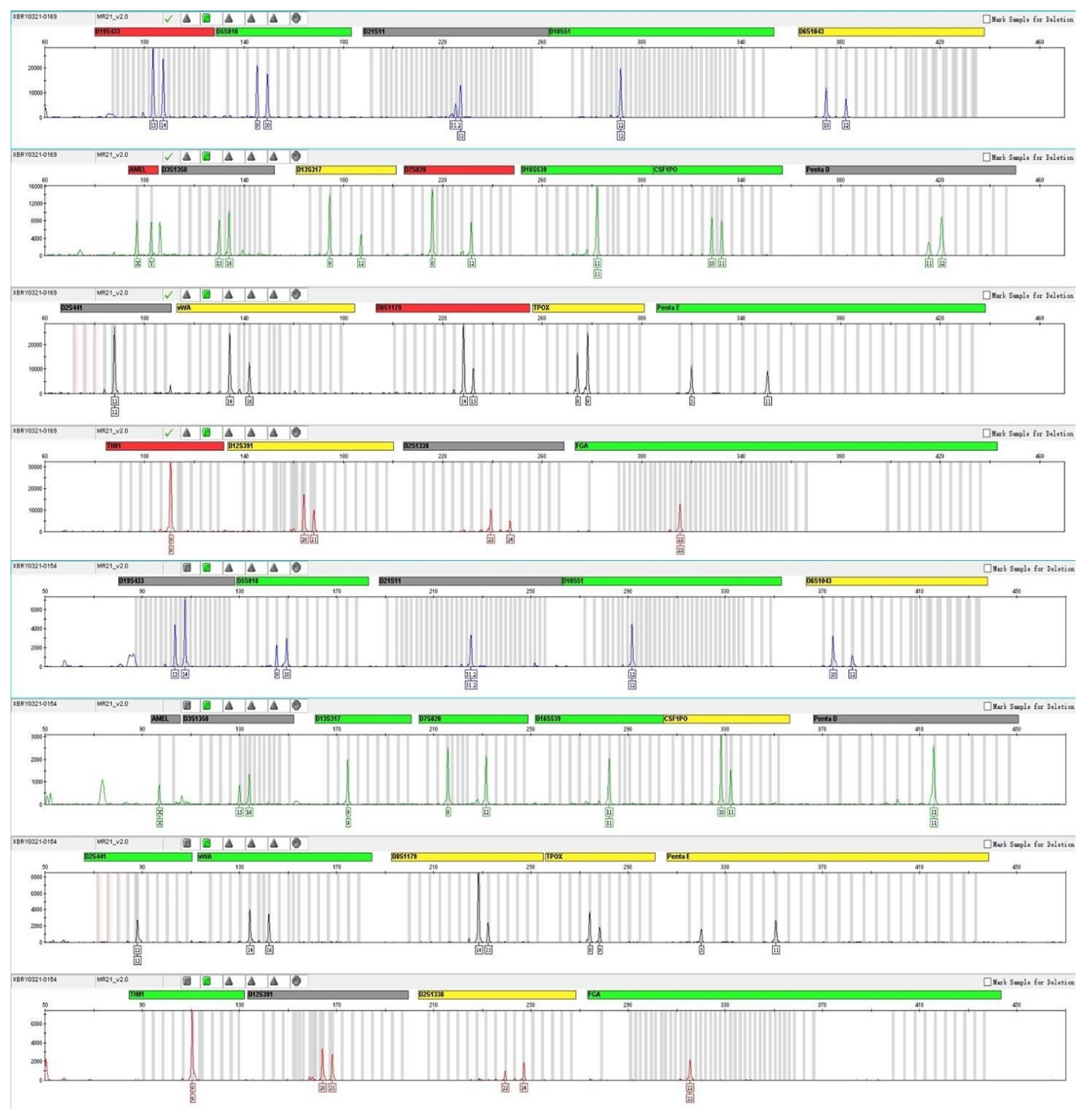

Supplement: Supplementary file 1 — Additional file 1. DNA fingerprinting of ICC-X1 cells. [file 12935_2022_2840_MOESM1_ESM.jpg]

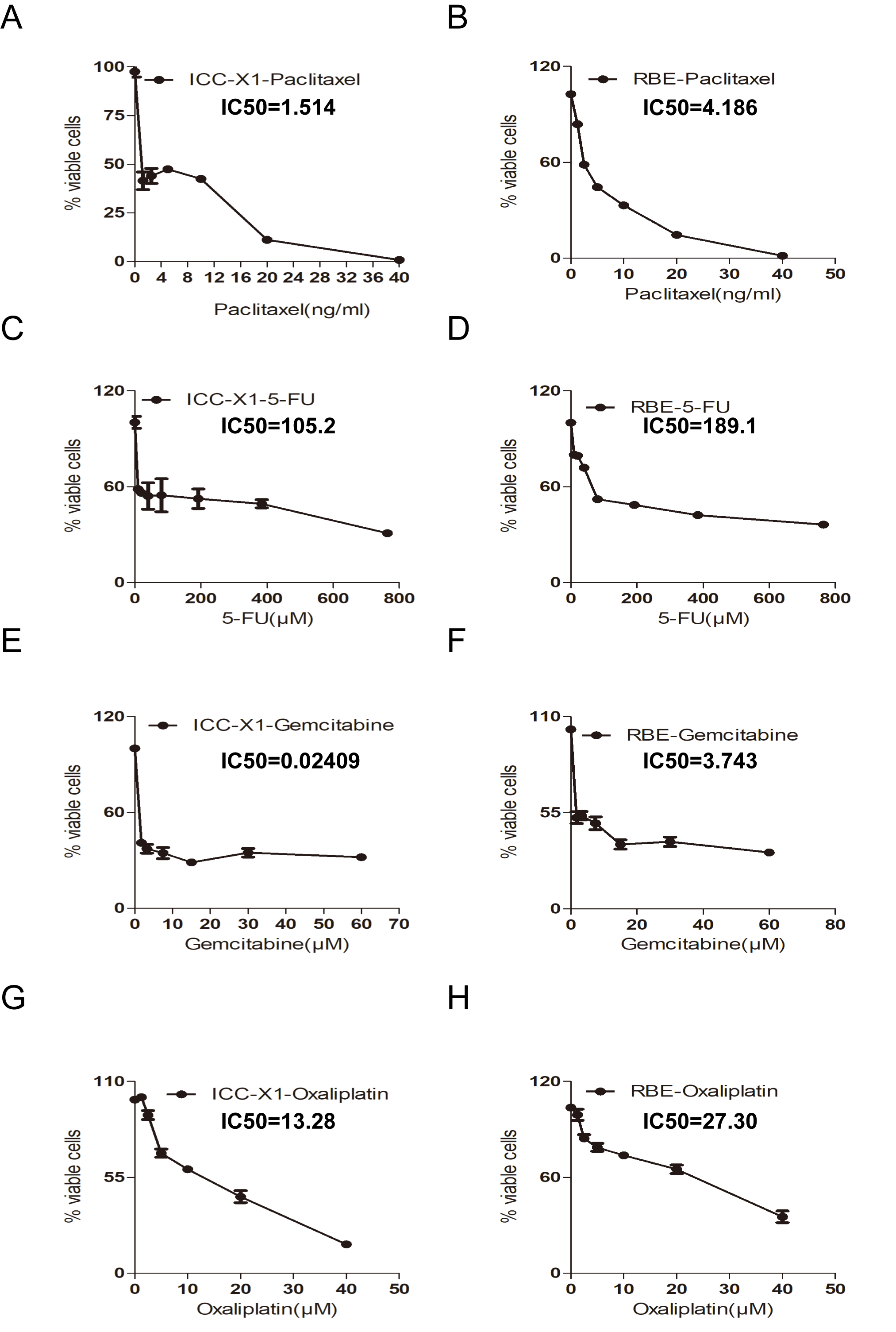

Supplement: Supplementary file 2 — Additional file 2. The dose-response curve of ICC-X1. [file 12935_2022_2840_MOESM2_ESM.tif]
